# Cannabinoids and the endocannabinoid system in immunotherapy: helpful or harmful?

**DOI:** 10.3389/fonc.2023.1296906

**Published:** 2023-11-22

**Authors:** Arailym Sarsembayeva, Rudolf Schicho

**Affiliations:** ^1^ Division of Pharmacology, Otto Loewi Research Center, Medical University of Graz, Graz, Austria; ^2^ BioTechMed, Graz, Austria

**Keywords:** cannabis, cannabinoid receptors, tumor microenvironment, endocannabinoid system, immune checkpoint inhibitor

## Abstract

Numerous studies in various cancer models have demonstrated that ingredients of cannabis can influence tumor growth through the endocannabinoid system (ECS), a network of molecules (mediators, receptors, transporters, enzymes) that maintains homeostasis and protection in many tissues. The main constituents of the ECS are the classical cannabinoid (CB) receptors, such as CB_1_ and CB_2_, their endogenous ligands (endocannabinoids), and the endocannabinoids’ synthesizing and degrading enzymes. The role of the ECS in cancer is still unclear and its effects often depend on the tumor entity and the expression levels of CB receptors. Many studies have highlighted the tumor cell-killing potential of CB_1_ agonists. However, cannabis is also known as an immunosuppressant and some data suggest that the use of cannabis during immunotherapy worsens treatment outcomes in cancer patients. CB receptors are widely present in immune cells, and together with monoacylglycerol lipase, the 2-arachidonoylglycerol degrading enzyme, they could be critically involved in the regulation of the immune cell profile of the tumor microenvironment (TME), and hence in tumor progression. So far, data on the impact of the ECS in the immune-TME are still vague. In this review, we discuss the current understanding of the ECS on immunoregulation during tumor growth, and how it might affect the outcome of cancer immunotherapy.

## Introduction

In ancient medicine, Chinese and Indian cultures widely used cannabis (derived from the plant *Cannabis sativa* L., mostly known as “marijuana”) for the treatment of various ailments (1). In the 1850s, cannabis was introduced into Western medicine by William B. O’Shaughnessy and Jacques-Joseph Moreau, who described its beneficial effects in treating rheumatism, convulsions, muscular spasms during tetanus and rabies, mental illnesses and cholera (2, 3). At the beginning of the 20^th^ century, the therapeutic use of cannabis considerably declined due to its abuse potential and the variability in the herbal material’s quality. At the same time, its use as a recreational drug increased. Thus, legal restrictions on the use of cannabis were imposed that delayed exploring its medical potential for more than 50 years (4–6). Only after the discovery of cannabinoid (CB) receptors in the early 1990s, attention was payed to this new field of research leading to the introduction of the endocannabinoid system (ECS). Since then, a huge number of studies have been conducted pertaining to the (patho)physiological roles of the ECS (7, 8).

The main constituents of the ECS are the classical CB receptors, CB_1_ and CB_2_, their endogenous ligands (endocannabinoids), such as anandamide (AEA) and 2-arachidonoylglycerol (2-AG), and the endocannabinoids’ synthesizing (diacylglycerol lipase (DAGL), N-acylphosphatidyl-ethanolamine phospholipase D (NAPE-PLD)) and degrading enzymes (fatty acid amide hydrolase (FAAH) and monoacylglycerol lipase (MAGL, also known as MGL)) (9, 10). CB_1_ is expressed throughout the body systems, predominantly in the central nervous system (abundant expression), but can be also found in the immune system (low expression) (9, 11). Factors that influence the expression of CB_1_ in immune cells are the type and activation status of the cells, the immune stimulus, and the presence of endocannabinoids (11, 12). CB_2_ is highly abundant in cells of the immune system, and its expression level is 10-100-fold higher than that of CB_1_ (11). B cells show the highest expression of the CB_2_ mRNA, followed by natural killer (NK) cells, monocytes, neutrophils, CD8^+^ T and CD4^+^ T cells (11). Many studies demonstrate that CB_1_ and CB_2_ expression may be either increased or decreased under pathological conditions, like in cancer, whereby the level of expression substantially influences tumor progression (13–17). Currently, the main indications for medical cannabis in cancer therapy are for palliative care to manage refractory nausea and pain, and to improve appetite (reviewed in (18)). The synthetic analogue of tetrahydrocannabinol (THC), nabilone (Cesamet), was licensed to suppress nausea and vomiting induced by chemotherapy. Moreover, dronabinol (Marinol), which is a synthetic THC, entered the clinic in 1985 as an anti-emetic, and in 1992 as an appetite stimulant (19).

## Importance of the ECS within the tumor microenvironment

The majority of *in vitro* studies on the ECS and cancer have focused on the direct activation or inhibition of CB receptors expressed in cancer cells, and the subsequent outcome on growth behavior (13–17). However, many questions about the impact of cannabinoids on immune cells in tumors, and hence on tumor progression, still remain unanswered.

Next to malignant cells, tumors consist of a heterogenous group of infiltrated and resident host cells, secreted factors and extracellular matrix, collectively called tumor microenvironment (TME). The composition of the TME changes between tumor entities, but distinctive characteristics of immune and stromal cells, blood vessels, and of the extracellular matrix remain. It is well established that TME cells actively participate in the regulation of cancer progression (20). Adaptive and innate immune cells are important components of the TME that either suppress or promote tumor growth. Within the TME, the adaptive immune response includes actions of T-, B-, and NK cells while the innate immune response works via the actions of myeloid cells like macrophages, neutrophils, eosinophils, dendritic cells (DCs), and other immune cells (20).

### Cannabinoids and the ECS: effect on the TME

Components of the ECS are virtually expressed in all cells of the tumor mass, including host cells of the TME as well as cancer cells, which makes it challenging to delineate their exact role in tumor development, and to use them as therapeutic targets. To date, only a handful of studies has looked at the influence of endo-/cannabinoids and ECS components on the TME, and their role in tumor development (reviewed in (21)). A summary of these studies and their outcomes are shown in **Table 1**. Recently, we reported that TME cell-derived CB_2_ promotes tumor growth in an experimental model of non-small cell lung cancer (NSCLC) by reducing accumulation and cytotoxic activity of CD8^+^ T and NK cells. We additionally observed, that deficiency of CB_2_ on host cells enhanced the expression of programmed death-1 (PD-1) and its ligand PD-L1 on lymphoid and myeloid cells, respectively (22). In the same NSCLC model, our group earlier identified that MGL expressed in TME cells was responsible for a pro-tumorigenic environment and modulated the immune cell profile (23). CD8^+^ T cells were increased in tumors of MGL knockout mice, and showed increased expression of granzyme-B, interferon-γ, and PD-1, signifying enhanced tumoricidal and immune activity (23). Since 2-AG also promotes the migration in these cells (23), MGL could be a major tumor driver in NSCLC. In a pancreatic cancer model, Qiu et al. showed that proliferation of tumors was compromised in the presence of 2-AG via activation of CB_1_, but not of CB_2_ (25). In this type of cancer, 2-AG promoted the maturation of DC phenotypes and the production of pro-inflammatory cytokines through up-regulation of the signal transducer and activator of transcription 6 (p-STAT6) (25). In a different study, THC had no direct effect on the growth of melanoma cells, but indirectly affected tumor growth via cells of the TME in a CB receptor-dependent manner (29). In particular, the infiltration of pro-tumorigenic myeloid immune cells was inhibited by THC, demonstrating that the ECS can interact with the TME in modulating tumor growth (29). In contrast to these studies, Xiang et al. demonstrated that MGL in macrophages inhibited CB_2_ receptor-dependent tumor progression. They described that MGL deficiency enhanced macrophage activation in a CB_2_/TLR4-dependent manner affecting the exhaustion status of CD8^+^ T cells in models of colon and breast cancer (26). In murine melanoma, absence of CB_2_ increased tumor growth and accumulation of immature B cells, reducing infiltration of CD8^+^ T cells into the TME (24).

**Table 1:** Influence of the ECS components and endocannabinoids and cannabinoids on the TME.

Cancer entity, year	ECS cannabinoid/endocannabinoid	Affected cells of the TME	Outcome	Ref.
**NSCLC, 2023**	CB_2_	CD8^+^ T and NK cells	CB_2_ plays a pro-tumorigenic role	(22)
**NSCLC, 2021**	MGL and 2-AG	Eosinophils, CD8^+^ T cells	MGL has a pro-tumorigenic role	(23)
**Melanoma, 2021**	CB_2_	B cells, CD8^+^ T cells	CB_2_ reduces tumor growth	(24)
**Pancreatic cancer, 2019**	2-AG and CB_1_	DCs and proinflammatory cytokines	2-AG inhibits proliferation of cancer cells via CB_1_	(25)
**Colon and breast cancer, 2018**	MGL and CB_2_	TAMs, CD8^+^ T cells	MGL influences cancer progression	(26)
**Colitis-associated colon cancer, 2020**	THC, CB_2_	DCs, macrophages, Tregs	THC attenuates colitis-associated cancer	(27)
**Lung cancer, 2016**	JWH-015	TAMs	CB_2_ agonist JWH-015 reduces tumor growth and decreases macrophage recruitment to tumor sites, decreases EMT of tumor cells	(28)
**Melanoma, 2015**	CB_1_ and CB_2_	CD45^+^ cells, Gr-1^-^ or Gr-1^+^ CD11b^+^ cells	THC reduces tumor growth in a CB receptor-dependent manner	(29)
**Breast cancer, 2015**	CBD	macrophages	CBD inhibits tumor growth and metastasis through inhibition of macrophage recruitment to tumor sites	(30)
**Glioma (C6), 2003**	JWH-133 and CB_2_	endothelial cells	CB_2_ agonist JWH-133 reduces tumor angiogenesis	(31)

2-AG, 2-arachidonoylglycerol; CB_1_, cannabinoid receptor 1; CB_2_, cannabinoid receptor 2; CBD, cannabidiol; DCs, dendritic cells; EMT, epithelial to mesenchymal transition; MGL, monoacylglycerol lipase; NK, natural killer; NSCLC, non-small cell lung cancer; TAMs, tumor-associated macrophages; THC, tetrahydrocannabinol; TME, tumor microenvironment. Tregs, regulatory T cells, JWH-015 and JWH-133, CB_2_ agonists.

Altogether, these reports suggest that components of the ECS like MGL and CB_2_ are able to affect tumor progression via immune cell activity and immune checkpoints of the TME. However, pro- or anti-tumorigenic effects of the ECS are likely dependent on the type of cancer.

## Immunotherapy and the ECS

CD8^+^ T cells work together with other immune cells of the TME to eradicate tumors. However, at some point during tumor development, immune cells, mainly cytotoxic CD8^+^ T cells, start losing their cytotoxic ability. Fortunately, targeted therapy has revolutionized the approach to treat cancer through the introduction of immunotherapy, which removes the brake from immune cells to eliminate tumors. Active (vaccines) and passive (monoclonal antibodies and adoptive cell therapy) therapies represent two subcategories of immunotherapy (32). Here, we briefly discuss how the ECS may interfere with the response to treatment with monoclonal antibodies, known as immune checkpoint inhibitors (ICIs).

In various types of tumors, the ECS has been shown to influence the immune cell composition of the TME and to subsequently affect immune cell activity and tumor growth (22–26, 29, 32). The activation and exhaustion status of immune cells, particularly of cytotoxic CD8^+^ T cells, are determined by the expression of immune checkpoint proteins, such as PD-1, cytotoxic T-lymphocyte antigen-4 (CTLA-4, aka CD152), and others like T cell immunoglobulin and ITIM domain (TIGIT), T-cell immunoglobulin and mucin-domain containing 3 (TIM-3), lymphocyte-activation gene 3 (LAG-3), and B- and T-lymphocyte attenuator (BTLA) (33). The main targets of current ICI therapies are PD-1/PD-L1 and the CTLA-4 receptor (34, 35). In 2011, a CTLA-4 blocking antibody (ipilimumab) was approved to treat melanoma. This was followed by the development of monoclonal antibodies targeting PD-1 (pembrolizumab and nivolumab) and PD-L1 (atezolizumab, durvalumab and avelumab). Furthermore, biomarkers to predict the efficacy of ICIs such as PD-L1 levels in tumor tissue, tumor mutation burden (TMB) and microsatellite instability (MSI) were introduced (36). PD-1/PD-L1 blocking antibodies have become standard treatment of various cancer types, including melanoma, NSCLC, mismatch repair-deficient and/or microsatellite-instable colorectal cancer, triple-negative breast cancer, head and neck squamous cell carcinoma, hepatocellular carcinoma, and several other cancers (37). The rate for effective treatment with anti-PD-1/PD-L1 antibodies varies with the type of tumor and lies around 80% in lymphoma, 60% in high MSI cancers, and approximately 10-30% in other common solid tumors (38). Owing to their reduced toxicity and their lower high-grade immune-related adverse events (irAEs), anti-PD-1/PD-L1 antibodies are used more often than anti-CTLA-4 antibodies (39–41).

Apart of the biomarkers that predict responsiveness to ICI therapy (36), other factors can cause resistance to immunotherapy, which must be taken into consideration before and during therapy. These include genomic factors, tumor heterogeneity, factors related to immune cells and the TME, factors emerging from host cell-cancer cell interactions, and other vital factors, such as advanced age, biological sex, diet, hormones, existing comorbidities, drugs, and the gut microbiome (42).

### Influence of cannabinoids and the ECS on immunotherapy

Immuno-oncologic studies aim to find ways to circumvent the development of resistance to immunotherapy and to determine adjuvant targets to increase patient survival. Because of its immunomodulatory effects, the ECS may hold such targets, but up to now, only few experimental or clinical studies have investigated the role of the ECS on the immune-TME and the impact of cannabis or cannabinoids in immunotherapy (shown in **Table 2**). Xiong and colleagues identified that exogenous (THC) as well as endogenous (AEA) cannabinoids negatively affect antitumor immunity by impairing the function of tumor-specific T cells via CB_2_. They also observed that THC reduces the therapeutic effect of anti-PD-1 therapy (43). In our NSCLC model, the response to anti-PD-1 treatment was improved in CB_2_ knockout mice suggesting that CB_2_ may act as an immunosuppressor in NSCLC. Additionally, anti-PD-1 antibody treatment increased the accumulation of cytotoxic immune cells (CD8^+^ T and NK cells) in CB_2_ knockout mice, suggesting that the presence of CB_2_ in the TME interferes with the response to PD-1/PD-L1 blockade (22). Targeting CB_2_ during immunotherapy could, therefore, have an additional benefit for NSCLC patients, and increase the response rate (22).

**Table 2:** The ECS and cannabinoids during immunotherapy.

Type of study	Cancer entity	ECS	ICIs	Outcome	Ref.
**Experimental mouse model**	Skin, colon, and lung cancers	AEA, CB_2_, THC	Anti-PD-1	THC reduces the therapeutic effect	(43)
**Experimental mouse model**	NSCLC	CB_2_	Anti-PD-1	The absence of CB_2_ favors a positive response	(22)
**Retrospective observational clinical study**	Advanced melanoma, NSCLC, and renal clear cell carcinoma	Cannabis	Anti-PD-1 (nivolumab)	The consumption of cannabis reduced RR, without influencing PFS and OS	(44)
**Prospective observational clinical study**	Melanoma, NSCLC, renal cell carcinoma, and other types of cancer	Cannabis	Anti-PD-1 (nivolumab, pembrolizumab) anti-CTLA-4 (ipilimumab), anti-PD-L1 (durvalumab, atezolizumab)	The consumption of cannabis before or during ICI immunotherapy associates with worsened clinical outcomes	(45)
**Experimental mouse model and retrospective non-randomized study**	Colorectal carcinoma (murine model, CT26 cell line), NSCLC (clinical study)	Cannabis	Anti-PD-1 (pembrolizumab) anti-PD-L1	No harmful effect of cannabis on the activity of pembrolizumab	(46)

AEA, anandamide; CB_2_, cannabinoid receptor 2; CTLA-4, cytotoxic T-lymphocyte antigen-4; ICIs, immune checkpoint inhibitors; NSCLC, non-small cell lung cancer; OS, overall survival; PFS, progression-free survival, PD-1, programmed death-1; PD-L1, programmed death-ligand 1; RR, response rate; THC, tetrahydrocannabinol.

Our experimental data largely fit with a clinical study, in which Taha et al. retrospectively demonstrated the effect of cannabis during immunotherapy (nivolumab) and evaluated the response rates (RR), progression-free survival (PFS), and overall survival (OS) of cancer patients. The group found that cannabis use during nivolumab therapy reduced RR without affecting PFS or OS (44). In a recent prospective observational clinical study, Bar-Sela et al. enrolled 102 advanced cancer patients (68 cannabis non-users and 34 cannabis users) who started ICI treatment. The findings showed that cannabis users had a significant decrease in time to treatment progression (TTP) and OS vs. cannabis non-users. The consumption of cannabis, however, reduced irAEs. The authors suggested to use cannabis with attentiveness before and during ICI immunotherapy of advanced malignances (45, 47). In contrast, a recent study by Waissengrin et al. described that concomitant use of medical cannabis with pembrolizumab had no harmful effect in advanced NSCLC. TTP of cannabis users did not differ from cannabis non-users. However, the median OS was numerically higher for cannabis non-users vs. cannabis users, but did not reach statistical difference (p = 0.08) (46).

Low response to ICI during cannabis use may be, therefore, linked to the fact that cannabinoids exert immunosuppressive effects (48). These effects may involve CB_2_ activity in immune or other TME cells.

## Discussion

Inflammation promotes tumorigenesis, but an active immune system is also a powerful weapon to combat tumor development. The fact that cells of the immune system are present in a shared microenvironment with cancer cells has been known since the 19^th^ century (49). However, not until recently, immune cells began to be used as tools against cancer, as Jennifer Couzin-Frankel wrote in *Science magazine*: *“*Immunotherapy marks an entirely different way of treating cancer- by targeting the immune system, not the tumor itself” (50). Today, many effective antibodies against checkpoint proteins are already at hand for antitumor treatment (37).

Downsides of immunotherapy include the development of resistance and a low responder rate (like in NSCLC) (51). Molecules other than checkpoint proteins like CB_2_ and MGL may, therefore, act as targets to aid the response to immunotherapy (22, 24–26, 29). CB_2_ is abundantly expressed in immune cells (52) and upon activation, it has the ability to influence immune functions, such as cytokine production, proliferation/differentiation, migration, apoptosis, and antibody production (reviewed in (12, 53). These functions are all amenable to CB_2_ receptor agonism/antagonism. Also MGL has been implicated as a driver in many types of tumors (23, 54, 55). A MGL inhibitor (ABX-1431) (56) has been already tested in the clinics (clinicaltrials.gov: NCT03625453). However, tumor type, the TME, or both seem to influence the behavior of CB_2_ and MGL towards tumors. This introduces an obstacle to the universal use of CB_2_ or MGL blockers for the treatment of cancer without prior knowledge of the ECS component’s role. Genetic modification of CB receptors in immune cells and adoptive cell therapy (e.g., CD8^+^ T and NK cells, known to express CB receptors (21)) could be one possible way of application. Investigating other members of the ECS, like DAGL, the 2-AG producing enzyme, and proteins involved in CB_2_ receptor signaling pathways would also be a step towards identifying new targets that could support ICI treatment.

It is known that co-medications such as proton pump inhibitors, glucocorticoids, antibiotics, psychotropic drugs and opioids must be carefully assessed at the time of ICI treatment initiation, as they may significantly change the antitumoral response (57–59) Likewise, the use of medical cannabis or cannabis-derived products during immunotherapy should be reconsidered as they might interfere with the ICIs’ mechanism. As cannabis use is widespread in cancer patients, ICI treatment may be unsuccessful if cannabis consumption is continued.

Further efforts are required to unravel the exact role of the ECS within the TME and to understand the importance of the ECS in tumor development and immunotherapy.

## Author contributions

AS: Writing – original draft, Writing – review & editing. RS: Writing – original draft, Writing – review & editing.
